# Determinants of non-recovery in physical health-related quality of life one year after cardiac surgery: a prospective single Centre observational study

**DOI:** 10.1186/s13019-020-01273-1

**Published:** 2020-09-01

**Authors:** Hilda Rijnhart-de Jong, Jo Haenen, Goris Bol Raap, Lilian Jekel, Tessel Vossenberg, Olga Bondarenko, Christiaan Boerma

**Affiliations:** 1 Hart- en Vaatcentrum, Leeuwarden Medical Centre, Henri Dunantweg 2, 8934 AD Leeuwarden, The Netherlands; 2 Departments of Intensive Care, Leeuwarden Medical Centre, Henri Dunantweg 2, 8934 AD Leeuwarden, The Netherlands

**Keywords:** Health-related quality of life, Cardiac surgery, Predictors, CABG, SF-36

## Abstract

**Background:**

Recent studies show that substantial percentage of patients experienced worsening of health related quality of life (HRQoL) 1 year after cardiac surgery. The aim of this study is to identify risk factors that interfere with improvement of HRQoL.

**Methods:**

From December 2015 till July 2017 a prospective single centre observational study was carried out in 1920 patients participated who underwent non-salvage cardiac surgery. All patients were requested to complete a Short Form 36 (SF-36) questionnaire before and 1 year after surgery. Primary aim of the study was to identify risk factors for non-recovery in the physical domain of the SF-36 in all cardiac surgery patients. Secondary aim was to identify identical risk factors in patients with isolated coronary artery bypass grafting.

**Results:**

After cardiac surgery, the questionnaires for physical and mental health were completed by respectively 803 and 807 patients. Median age was 69[62–75] years, and 77% was male. In comparison to the preoperative status, 176 patients (21.9%) did not display an improvement in the SF-36 physical domain score 1 year after cardiac surgery. In a multivariate analysis independent risk factors for non-recovery in the SF-36 physical domain were baseline SF36 physical domain score (OR 0.954[0.942–0.965], *P* < 0.001), diabetes (OR 0.437 [0.265–0.720], P 0.001), female sex (OR 0.492 [0.307–0.789], P 0.003), post-operative infection (OR 0.240 [0.109–0.525], *P* < 0.001) and PCI within 1 year (OR 0.113 [0.036–0.349], *P* < 0.001) For isolated CABG, 23.2% of patients did not display an improvement in the physical domain score and risk factors appeared to be identical.

**Conclusions:**

Twenty two percent of all cardiac surgery patients did not show an improvement in the physical domain score of the HRQoL between the preoperative period and 1 year after surgery. Independent risk factors for non-recovery after cardiac surgery were baseline SF-36 physical domain score, diabetes, female sex, any postoperative infection and the need for PCI in the first year. Further research is needed to tailor the patient selection procedure prior to surgery and potentially modify risk factors in the perioperative process.

**Trial registration:**

Due to type of study not applicable. https://www.ccmo.nl/metcs/erkende-metcs/regionale-toetsingscommissie-patientgebonden-onderzoek.

## Background

Traditionally, objective outcome measures, including survival, complication rates and need for re-interventions have been used to benchmark cardiac surgery. During the last decades additional assessment of health-related quality of life (HRQoL) scores have been used in the evaluation of cardiac interventions. Typically, HRQoL scores serve as secondary outcome parameters in the comparison of strategies related to coronary artery bypass grafting (CABG), e.g. on-pump versus off-pump techniques or CABG versus percutaneous coronary interventions (PCI) [1, 2]. Alternatively, studies focus on specific baseline characteristics, such as age, sex or severity of angina in relation to HRQoL-related outcome measures [3–7]. However, despite a well-established general improvement in HRQoL in various settings of cardiac surgery, a recent study underlined the observation that 20% of patients experienced worsening of HRQoL 1 year after CABG as compared to the preoperative status [8]. Although this is generally considered acceptable, such assessment may differ substantially from the perception of individual patients, especially in the setting of limited reduction in HRQoL prior to surgery [6]. The purpose of this study was to identify risk factors that interfere with improvement of HRQoL 1 year after cardiac surgery.

## Methods

### Setting and patient selection

In this prospective single-centre observational study all non-salvage patients, scheduled for a cardiothoracic surgical procedure during December 2015 till July 2017 were eligible for inclusion. All collected data were part of a nationwide Dutch Heart Registry, that aims to evaluate and promote quality of all cardiovascular interventions in the Netherlands [9]. A local hospital ethics committee (Regionale Toetsingscommissie Patiëntgebonden Onderzoek, Leeuwarden, the Netherlands) waived the need for additional informed consent (nWMO 2,020,004). Preoperative, intraoperative- and postoperative data with a 1-year follow-up were collected and stored in a pseudonymized database.

### Measurement of the HRQL and definitions

Prior to surgery and 1 year after surgery, all eligible patients were asked to fill in a complete Short Form (SF)-36 general health status survey (version 2). Questionnaires were handed-out in the outpatient clinic for elective surgery patients and in the clinical setting for urgency/emergency surgery patients. In case a minimum of 50% of the questions was answered in each domain of both preoperative and postoperative questionnaires a patient was classified as responder. The SF-36 is a standardized HRQoL assessment tool, originally described by Ware et al. and additionally validated in the setting of coronary artery disease [10, 11]. It consists of 36 multiple choice questionnaires divided over eight domains: physical function (PF), role limitations due to physical problems (RP), body pain (BP), general health perception (GH), vitality (VT), social functioning (SF), role limitations due to emotional problems (RE) and mental health (MH). Grading scores range from 0 to 100. The individual scores of all physical domains are combined and expressed as SF-36 physical health component; individual scores of all mental domains are combined and expressed as SF-36 mental health component [10].

Patients were separated into two groups according to the change in SF-36 physical domain scores over time. One group consisted of patients with an improvement in physical domain score from the pre-operative period to 1 year after surgery (recovery (R) group). The other group consisted of patients with a reduction or equal physical score from the pre-operative period to 1 year after surgery (non-recovery (NR) group). Infection was defined as every registered infection, according to predefined criteria [9], irrespective of site or severity. Urgent surgery was defined as the require for surgery, not electively admitted, and needed on the current admission for medical reasons. Emergency was defined as operation before the beginning of the next working day after decision to operate. Readmission is scored with the first 30 days after hospital discharge.

### Statistical analyses

Data analyses were performed using the statistical package for social science (SPSS 24 for Windows; SPSS INC, Chicago, IL). Data distribution for continuous variables was tested by means of a Kolmogorov-Smirnov test. In case of non-normal distribution, data are presented as median [interquartile range, IQR] or as mean ± standard deviation (SD in case of normal distribution. Comparison of continuous data between groups was performed by a non-parametric Mann-Whitney U test, and in case of categorical data by Chi-square testing. Univariate analysis was performed using the log rank test. All (sub) items of the EuroSCORE II were included separately, specifically avoiding repetition of baseline characteristics. Variables with a *p*-value of < 0.25 in the univariate analysis were included in the bivariate logistic regression analysis. Using a backward likelihood ratio selection, variables associated with the NR group were determined. Odds ratios (OR) with 95% confidence intervals (CI) were presented for all variables in the final model. *P*-values < 0.05 were considered statistically significant.

Primary endpoint of the study was to identify differences in characteristics between the R-group and the NR-group in the overall cohort of mixed cardiac surgery patients. Secondary endpoint was to identify differences in characteristics between the R-group and NR-group of isolated CAGB patients. Similar endpoints for (non)recovery with respect to the SF-36 mental health domain are presented as electronic supplemental material (ESM).

## Results

### Primary endpoint (all cardiac surgery patients)

#### Baseline characteristics

**Fig. 1:**
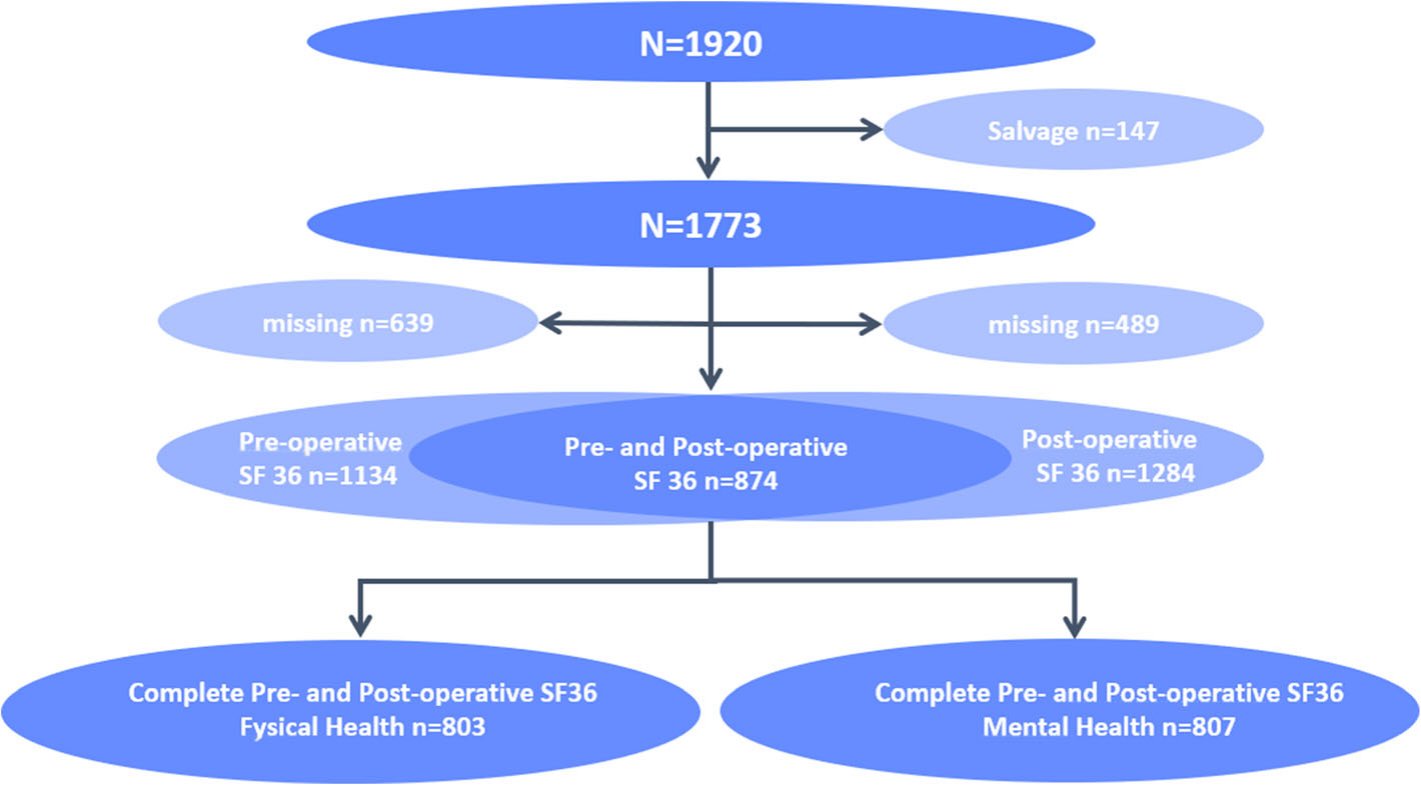
Flow chart of eligible patients

A total of 803 out of 1773 eligible patients (45%) completed the SF-36 on both time points and were included in the study (Fig. 1). In this group of responders the minimum percentage of completed questions was 95%. Median age was 69[62–75] years, 77% was male (Table 1). In comparison to the preoperative status, 176 patients (21.9%) did not display an improvement in the SF-36 physical domain score 1 year after cardiac surgery. At baseline the median SF 36 physical domain score was significantly lower in the R-group (56[42–69] vs. 71[55–86], *p* < 0.001). In a univariate analysis pre-operative characteristics, including individual components of the European System for Cardiac Operative Risk Evaluation (EuroSCORE) II risk model, did not differ between the R-group and the NR-group, with the exception of a recent myocardial infarction, which was more often present in NR patients (27.8 vs. 19.6%, *p* = 0.019). However, total EuroSCORE II was higher in the NR-group (1.8[1.2–3.0] vs. 1.5[1.0–2.6], *p* = 0.03).

**Table 1:** Characteristics of all cardiac surgery patients in the physical recovery group and non-recovery group

	All (*n* = 803)	R (*n* = 627)	NR (*n* = 176)	*p*-value
**Demographics**				
Baseline SF 36 physical domain score	59 [45–73]	56 [42–69]	71 [55–86]	< 0.001^a^
Body Mass Index (kg/m^2^)	27 [25–30]	27 [25–30]	27 [25–29]	0.891
**Comorbidities**				
CVA (%)	2	1.6	1.1	0.658
Neurologic dysfunction (%)	2	2.1	1.7	0.757
**Cardiac status**				
Unstable AP (%)	3.5	3.2	4.5	0.387
**EuroSCORE II**	1.6 [1.1–2.7]	1.5 [1.0–2.6]	1.8 [1.2–3.0]	0.030^a^
*Age (years)*	69 [62–75]	69 [62–75]	70 [62–75]	0.127
*Gender Male (%)*	77	78	73	0.144
*Serum Creatinine (umol/l)*	84 [73–96]	84 [73–96]	82 [72–95]	0.347
*Extracardiac arteriopathy (%)*	8	7.5	9.1	0.487
*Poor mobility (%)*	2.0	2.1	1.7	0.757
*Previous cardiac surgery (%)*	3.1	3.0	3.4	0.798
*Chronic lung disease (%)*	12	11.6	10.8	0.755
*Active endocarditis (%)*	0.7	0.6	1.1	0.498
*Critical preoperative state (%)*	0.5	0.3	1.1	0.174
*Diabetes (%)*	18.9	17.5	23.9	0.059
*NYHA Class III or IV (%)*	54	53.5	55.1	0.692
*Angina CCS Class IV (%)*	1.9	2.2	0.6	0.150
*LVEF (%)*				0.254
*Good*	77	78	75	
*Moderate*	18	17	22	
*Poor*	4	4	2	
*Missing*	1	1	1	
*Recent MI (%)*	21	19.6	27.8	0.019^a^
*Pulmonary hypertension (%)*	12.6	12.8	11.9	0.654
*Urgency (%)*				0.231
*Elective*	50	51.4	45.5	
*Urgent*	48.7	47.5	52.3	
*Emergency*	1.3	1.1	2.3	
*Weight of intervention (%)*				0.456
*Isolated CABG*	69.4	68.6	72.2	
*Single Non CABG*	13.1	13.1	13.1	
*2 Procedures*	15.4	16.4	11.9	
*3 Procedures*	2.1	1.9	2.8	
*Surgery on thoracic aorta (%)*	2.7	3.0	1.7	0.341
**Intraoperative characteristics**				
Aortic cross-clamp (min)	60 [42–83]	61 [42–83]	59 [44–81]	0.358
ECC (min)	91 [64–118]	92 [63–119]	86 [64–116]	0.382
**Postoperative characteristics**				
Infection (%)	5.6	4.5	9.7	0.008^a^
First detubation (hour)	3.2 [2.5–4.7]	3.2 [2.5–4.6]	3.2 [2.5–4.9]	0.868
Re-sternotomy < 30 d (%)	4.1	4.0	4.0	0.765
ICU days (n)	1.0 [1.0–1.0]	1.0 [1.0–1.0]	1.0 [1.0–1.0]	0.450
ICU stay extended (%)	5.9	5.9	5.7	0.913
CVA (%)	0.3	0.2	0.6	0.336
Readmission ICU (%)	1.0	0.8	1.7	0.285
Readmission < 30 days after discharge (%)	0.9	0.5	2.3	0.026^a^
MI (incl. Perioperative MI) (%)	3.1	3.2	2.8	0.740
MI (excl. Perioperative MI) (%)	0.9	0.6	1.7	0.142
PCI < 1 year (%)	2.2	1.3	5.7	< 0.001^a^

Abbreviations: *R* recovery group, *NR* non-recovery group, *CVA* cerebral vascular accident, *AP* angina pectoris, *EuroSCORE* European System for Cardiac Operative Risk Evaluation, *NYHA* New York Heart Association, *CCS Class 4 Angina* Inability to perform any activity without angina or angina at rest, *LVEF* left ventricular ejection fraction, *MI* Myocardial infarction, *CABG* coronary artery bypass grafting, *ECC* extracorporeal circulation, *ICU* intensive care unit, *PCI* percutaneous coronary intervention

^a^Indicates a significant difference across groups. Data are presented as median [interquartile range] unless stated otherwise

#### Intraoperative and postoperative characteristics

Type and duration of surgery, as well as the percentage of non-elective procedures did not significantly differ between groups (Table 1). In the postoperative phase significantly more patients in the NR group had infective complications (9.7 vs. 4.5%, *p* = 0.008), higher readmission rates (2.3 vs. 0.5%, *p* = 0.026) and higher percentages of PCI re-interventions (5.7 vs. 1.3%, *p* < 0.001).

#### Multivariate analysis of all cardiac surgery patients

Binary logistic regression analysis identified three independent preoperative risk factors for non-recovery of physical health: baseline SF36 physical component score, diabetes and female sex. In addition, two postoperative risk factors were identified: infections and PCI re-intervention within 1 year (Table 2). None of the intraoperative characteristics, including type of surgery, were independently associated with non-recovery. The pseudo R-square indicates that the model explains 23% of the observed variation.

**Table 2:** Multivariate analysis of all cardiac surgery patients. Dependent variable: physical recovery

	*p*-value	OR	95% CI
Baseline SF36 physical domain score	< 0.001	0.954	0.942–0.965
Diabetes	0.001	0.437	0.265–0.720
Female sex	0.003	0.492	0.307–0.789
Infection	< 0.001	0.240	0.109–0.525
PCI < 1 year	< 0.001	0.113	0.036–0.349

Hosmer and Lemeshow X^2^ = 8.97, *p* = 0.345; Nagelkerke *R*^*2*^ = 0.228.

### Secondary endpoint (isolated CABG patients)

#### Baseline characteristics

A total of 555 out of 1206 (46%) isolated CABG patients completed the full SF-36 on both time points and were included in the analysis. Median age was 68[62–74] years, 84% was male (Table 3). In comparison to the preoperative status, 129 patients (23.2%) did not display an improvement in the SF-36 physical domain score 1 year after cardiac surgery. At baseline the median SF-36 physical domain score was significantly lower in the R-group (56[44–69] vs. 73[57–88], *p* < 0.001). In a univariate analysis pre-operative characteristics did not differ between groups, with the exception of a recent myocardial infarction, which was more often present in the NR-group (38 vs. 26.5%, *p* = 0.012) and female sex which was more often present in the NR-group (24 vs 14%, *p* = 0.003). However, total EuroSCORE II was higher in the NR-group (1.7[1.1–2.5] vs. 1.3[0.9–2.0], *p* = 0.021).

**Table 3:** Characteristics of isolated CABG patients in the physical recovery group and non-recovery group

	All (*n* = 555)	R (*n* = 426)	NR (*n* = 129)	*p*-value
**Demographics**				
Baseline SF 36 physical domain score	60 [46–73]	56 [44–69]	73 [57–88]	< 0.001^a^
Body Mass Index (kg/m^2^)	27.1 [24.8–29.4]	27.0 [24.8–29.6]	27.2 [24.8–29.1]	0.728
**Comorbidities**				
CVA (%)	0.7	0.9	0.0	0.270
Neurologic dysfunction (%)	1.1	1.2	0.8	0.702
**Cardiac status**				
Unstable AP (%)	4.9	4.5	6.2	0.421
**EuroSCORE II**	1.37 [0.98–2.12]	1.3 [0.9–2.0]	1.7 [1.1–2.5]	0.021^a^
*Age (years)*	68.4 [61.8–74.0]	67.9 [61.7–73.7]	69.5 [62.2–74.4]	0.204
*Gender Male (%)*	83.6	85.9	76.0	0.003^a^
*Serum Creatinine (umol/l)*	84 [24.8–29.4]	84 [75–95]	82 [72–95]	0.762
*Extracardiac arteriopathy (%)*	8.1	7.5	10.1	0.350
*Poor mobility (%)*	1.1	1.2	0.8	0.702
*Previous cardiac surgery (%)*	1.3	1.4	0.8	0.573
*Chronic lung disease (%)*	10.6	10.6	10.9	0.926
*Active endocarditis (%)*	0.0	0.0	0.0	1.000
*Critical preoperative state (%)*	0.5	0.2	1.6	0.074
*Diabetes (%)*	20.2	18.8	24.8	0.135
*NYHA Class III or IV (%)*	57.1	55.9	61.3	0.281
*Angina CCS Class IV (%)*	2.3	2.8	0.8	0.180
*LVEF (%)*				0.116
*Good*	78.9	81.0	72.1	
*Moderate*	17.5	15.7	23.3	
*Poor*	2.7	2.6	3.1	
*Missing*	0.9	0.7	1.6	
*Recent MI (%)*	29.2	26.5	38.0	0.012^a^
*Pulmonary hypertension (%)*	1.6	1.6	1.6	0.942
*Urgency (%)*				0.268
*Elective*	39.1	40.8	33.3	
*Urgent*	59.3	58.0	63.6	
*Emergency*	1.6	1.2	3.1	
*Weight of intervention (%)*				
*Isolated CABG*	99.3	99.5	98.4	0.452
*Single Non CABG*	0.0	0.0	0.0	
*2 Procedures*	0.5	0.2	1.6	
*3 Procedures*	0.2	0.2	0.0	
*Surgery on thoracic aorta (%)*	0.2	0.0	0.8	0.069
**Intraoperative characteristics**				
Aortic cross-clamp (min)	51.0 [37.0–70.0]	51 [37–70.3]]	52 [37–70.5]	0.479
ECC (min)	80.0 [56.0–103.0]	80 [55–103]	81 [58.5–105.5]	0.343
**Postoperative characteristics**				
Infection (%)	5.0	3.5	10.1	0.007^a^
First detubation (hour)	3.0 [2.5–4.4]	3.0 [2.5–4.3]	3.1 [2.5–4.9]	0.412
Re-sternotomy < 30 d (%)	3.8	4.0	3.1	0.750
ICU days (n)	1.0 [1.0–1.0]	1.0 [1.0–1.0]	1.0 [1.0–1.0]	0.666
ICU stay extended (%)	2.9	3.1	2.3	0.666
CVA (%)	0.2	0	0.8	0.180
Readmission ICU (%)	0.5	0.5	1.6	0.889
Readmission < 30 days after discharge (%)	1.1	0.5	3.1	0.042^a^
MI (incl. perioperative) (%)	3.6	3.8	3.1	0.739
MI (excl. perioperative) (%)	0.9	0.7	1.6	0.699
PCI < 1 yr (%)	2.9	1.4	7.8	0.001^a^

Abbreviations: *R* recovery group, *NR* non-recovery group, *CVA* cerebral vascular accident, *AP* angina pectoris, *EuroSCORE* European System for Cardiac Operative Risk Evaluation, *NYHA* New York Heart Association, *CCS Class 4 Angina* Inability to perform any activity without angina or angina at rest, *LVEF* left ventricular ejection fraction, *MI* Myocardial infarction, *CABG* coronary artery bypass grafting, *ECC* Extracorporeal circulation, *ICU* intensive care unit, *PCI* percutaneous coronary intervention

^a^Indicates a significant difference across groups. Data are presented as median [interquartile range] unless stated otherwise

#### Intraoperative and postoperative characteristics

Duration of surgery and the percentage of non-elective procedures did not significantly differ between groups (Table 3). In the postoperative phase significantly more patients in the NR-group had infective complications (10.1 vs. 3.5%, *p* = 0.007), higher readmission rates (3.1 vs. 0.5%, *p* = 0.042) and higher percentages of PCI re-interventions (7.8 vs. 1.4%, *p* = 0.001).

#### Multivariate analysis of isolated CABG patients

Binary logistic regression analysis identified three independent preoperative risk factors for non-recovery of physical health: baseline SF-36 physical domain score, diabetes and female sex. In addition, two postoperative risk factors were identified: infections and PCI re-intervention within 1 year (Table 4). None of the intraoperative characteristics, including type of surgery, were independently associated with non-recovery. The pseudo R-square indicates that the model explains 26% of the observed variation.

**Table 4:** Multivariate analysis of isolated CABG patients. Dependent variable: physical recovery

	*p*-value	OR	95% CI
Baseline SF36 physical domain score	< 0.001	0.954	0.942–0.967
Diabetes	0.004	0.443	0.257–0.766
Female sex	< 0.001	0.341	0.194–0.601
Infection	< 0.001	0.195	0.078–0.488
PCI < 1 year	< 0.001	0.106	0.032–0.353

Hosmer and Lemeshow X^2^ = 10.9, *p* = 0.209; Nagelkerke *R*^*2*^ = 0.255.

#### Electronic supplemental material (ESM)

Additional analyses of patient characteristics for (non)recovery with respect to the SF-36 mental domain, information on the interplay between physical and mental recovery, data on the characteristics of non-responders and the CABG flowchart are provided in the ESM [see Additional file 1].

## Discussion

In the present study 21.9% of all cardiac surgery patients did not show an improvement in the physical domain score of the SF-36 HRQoL between the preoperative period and 1 year after surgery. Baseline SF-36 physical domain score, diabetes, female sex, any postoperative infection and the need for PCI in the first year were identified as independent risk factors for non-recovery. Selection of isolated CABG patients did not change this picture. An important finding in our study is the lack of predictive power of the currently used EuroSCORE II risk model with respect to physical recovery. Specifically designed and calibrated for the prediction of short-term mortality, the predictive power of the EuroSCORE II risk model is known to diminish with respect to 1 and 5 year mortality [12, 13]. Notwithstanding the fact that other risk models claim better performance for long-term mortality, the relationship between such risk scores and HRQoL-related endpoints remains largely unexplored [14, 15].

At first glance our findings seem to contradict the existing literature. After all, cardiac surgery including CABG, is associated with an improvement in even very long-term HRQoL [7, 16, 17]. A hallmark of these papers is a comparison in HRQoL over time for a substantial cohort of patients. However, such general improvement does not rule out the existence of subgroups of patients that do not benefit from surgical intervention. Recently, a reduction in the physical component scale of the Veterans Rand (VR)-36 questionnaire was observed in 13.7% of CABG patients 1 year after surgery, despite a general improvement in the total population [8]. In addition, in 39.7% of the patients physical performance remained unchanged over time. Similarly, a general improvement in HRQoL up till 18 months was observed in a cohort of CABG patients. However, the authors highlighted the fact that they also observed an age-dependent decline in HRQoL in patients > 75 years of age between 6 and 18 months after surgery [18]. In a more selected group of high-risk patients with an indication for either surgical aortic valve replacement or transcatheter aortic valve implantation, an absence in increased physical performance at 6-months was as high as 58.7% [19]. Our data do not confirm age as independent risk factor, as previously reported in the setting of elective CABG and aortic valve replacement [20, 21]. This may be due to differences in inclusion criteria (our study also reports on non-elective surgery patients) or the fact that age was included in our multivariate model as a continuous, rather than a categorical variable.

In general, comparison of our findings with the existing literature on HRQoL after cardiac surgery is hampered by a the fact that many papers have a retrospective design, lack baseline values and use different HRQoL grading system [4]. Our data confirm a previous publication in which diabetes was also identified as an independent risk factor for deterioration in VR-36 derived physical performance prior to and 1 year after surgery [8]. However, others observed a similar improvement in overall HRQoL 5 years after CABG in diabetic and non-diabetic patients, despite lower baseline values [22]. Among multiple factors, both diabetes and female sex appeared to be independent risk factors for non-improvement in HRQoL in a cohort of CABG patients [23]. The observation of female sex as a specific determinant for worsening HRQoL after 6 months and 10 years respectively has been confirmed by others [5, 17, 22]. To the analogy of our observations, baseline HRQoL as an independent marker of improvement in HRQoL was additionally identified in large cohort of CABG patients with a 10 years follow up. Risk factors for non-recovery beyond baseline characteristics are scarcely studied. In a mixed cardiac surgery group prolonged length of stay (> 2 days) in the Intensive Care Unit was associated with reduced recovery in SF-36 scores as compared to propensity matched controls [16]. Although we were unable to replicate these data, it is clear that the observed influence of any infection during hospital stay is likely to reflect morbidity from a different perspective.

We believe our data may have profound implications for the way we inform patients prior to surgery. The inability of commonly applied risk models to predict physical non-recovery adequately urges us to extend our knowledge over the full chain of events in the preoperative, perioperative and postoperative setting. The fact that preoperative physical performance is strongly related to the patient’s ability to improve (i.e. the odds for non-recovery increased roughly 5% for each increase in baseline SF-36 physical domain score by one point) seems to make sense from an analytical perspective. However, from the patient’s perspective this may be very relevant. If the aim of the surgical procedure is to improve the physical performance of the patient, an unsatisfactory result becomes more likely in case baseline physical performance is only mildly affected. In addition, women seem less likely to improve in comparison to men. If so, it is not only fair to incorporate such wisdom in the way we obtain informed consent, but it also necessitates further research to unravel sex-related mechanisms beyond the acute coronary syndrome [24]. Efforts to reduce infections in the postoperative phase seem worthwhile and such knowledge may help to motivate staff for better compliance with infection prevention bundles. Finally, the possibility of recurrence of symptoms that fuelled the process towards a surgical procedure in the first place (i.e. angina), represented by the need for PCI within 1 year after surgery, may deserve more attention in our communication with the patient. In addition, further research to unravel underlying mechanisms, including the interplay between recurrence of angina with perioperative infections, is needed [25].

The current study has several limitations. The single centre design does not allow to extend our conclusions beyond the specific setting. Despite the prospective design, including assessment of baseline HRQoL, the amount of missing data is substantial. Furthermore, we deliberately chose to restrict our analysis to the physical performance domain of the SF-36, were others suggest to combine different domains of HRQoL in order to provide additional HRQoL information. However, multivariate models with multiple endpoints become very difficult to interpret and suffer from statistical power, even in a substantial cohort with over 800 patients. Instead we chose to present data on the (non)recovery of mental HRQoL, and its overlap with physical HRQoL in the ESM. In general, our study fully complies with 8 out of 10 recommended quality criteria for HRQoL research in the specific field of cardiac surgery [26]. Furthermore we were unable to incorporate frailty in our model, which may have yielded additional information [27, 28]. Overlap with items such as ‘poor mobility’ that characterizes vitality to some extent remain to be established. Although the response rate of 45% is generally considered acceptable in this field of research, we have to acknowledge the fact that the substantial part of the population was not included. From the ESM Table 5 it becomes clear that non-responders were in general significantly more at risk. However how this translates to HRQoL remains to be established. Finally, it must be noted that pseudo R-squares of our multivariate models (0.23 for all patients and 0.26 for CABG) indicate that yet unnoticed factors may be of influence outside the scope of our analysis. This should fuel further research that incorporates other factors yet unaccounted for, such as social status, feeding and rehabilitation programs.

## Conclusion

One year after cardiac surgery 22 % of all cardiac surgery patients experienced a decrease in physical HRQoL. Baseline physical domain score, diabetes, female sex, any postoperative infection and the need for PCI in the first year were identified as independent risk factors for non-recovery. Further research is needed to tailor the patient selection procedure prior to surgery and potentially modify risk factors in the perioperative process.

## Supplementary information

**Additional file 1 Table 1.** Characteristics of all cardiac surgery patients in the mental recovery group and non-recovery group. **Table 2.** Multivariate analysis of all cardiac surgery patients. Dependent variable: mental recovery. **Table 3.** Characteristics of isolated CABG patients in the mental recovery group and non-recovery group. **Table 4.** Multivariate analysis of isolated CABG patients. Dependent variable: mental recovery. **Table 5.** Characteristics of all cardiac surgery patients in the Responders and non-Responders group. **Figure 1.** Flow chart of CABG patients. **Figure 2.** Venn diagram of all patients. Interplay between changes in SF-36 physical and mental domain scores prior to and 1 year after surgery (%). **Figure 3.** Venn diagram of isolated CABG patients. Interplay between changes in SF-36 physical and mental domain scores prior to and 1 year after surgery.

## Data Availability

The datasets used and/or analysed during the current study are available from the corresponding author on reasonable request.

## Abbreviations

HRQoL: Health related quality of life
CABG: Coronary Artery Bypass Grafting
PCI: Percutaneous Coronary Intervention
SF-36: Short Form 36
PF: Physical function
RP: Role limitations due to physical problems
BP: Body pain
GH: General Health Perception
VT: Vitality
SF: Social functioning
RE: Role limitations due to emotional problems
MH: Mental Health
SD: Standard deviation
EuroSCORE II: European System for Cardiac Operative Risk Evaluation
LVEF: Left ventricular ejection fraction
CVA: Cerebral vascular accident
AP: Angina Pectoris
NYHA: New York Heart Association
CCS Class IV: Inability to perform any activity without angina or angina at rest
MI: Myocardial infarction
ECC: Extracorporeal circulation
ICU: Intensive Care Unit
OR: Odds ratio
CI: Confidence Interval
