# Supplementary material for: Determinants of non-recovery in physical health-related quality of life one year after cardiac surgery: a prospective single Centre observational study
Source: J Cardiothorac Surg. 2020 Sep 1;15:234. doi: 10.1186/s13019-020-01273-1 (PMC7466488; doi:10.1186/s13019-020-01273-1)

ESM Table 1. Characteristics of all cardiac surgery patients in the mental recovery group and non-recovery group.

	All (n=807)	R (n=574)	NR (n=233)	p-value
**Demographics**				
Baseline SF 36 mental domain score	68 [54-81]	65 [49-77]	78 [67-89]	<0.001*
Body Mass Index (kg/m^2)	27 [25-30]	26.9 [24.68-29.43]	27.1 [24.8-29.65]	0.503
**Comorbidities**				
CVA (%)	1.5	1.6	1.3	0.766
Neurologic dysfunction (%)	1.9	1.9	1.7	0.849
**Cardiac status**				
Unstable AP*(%)*	3.5	3.3	3.9	0.698
**EuroSCORE II**	1.6 [1.1-2.7]	1.5 [1.0-2.6]	1.7 [1.1-2.9]	0.118
Age (%)	68.9 [62.4-75.1]	69 [62.85-75.1]	68.8 [61.84-75.1]	0.776
Gender male (%)	76	76.8	74.2	0.436
Creatinine clearance (umol/l)	84 [73-96]	84 [74-96]	82 [72-96]	0.251
Extracardiac arteriopathy (%)	7.6	7.0	9.0	0.320
Poor Mobility (%)(%)	1.9	1.9	1.7	0.849
Previous cardiac surgery (%)	3.2	3.0	3.9	0.512
Chronic lung Disease (%)	11.0	11.5	9.9	0.504
Active endocarditis (%)	0.9	0.9	0.9	0.986
Critical pre-operative state (%)	0.5	0.5	0.4	0.864
Diabetes (%)	18.5	17.9	19.7	0.551
NYHA Class III or IV (%)	54	51	59.7	0.026*
Angina CCS Class IV (%)	2.0	1.9	2.1	0.832
LVEF (%)				0.683
Good	77.2	77.7	76	
Moderate	18.2	17.4	20.2	
Poor	3.6	3.7	3.4	
missing	1.0	1.2	0.4	
Recent MI (%)	21.7	17.9	30.9	0.001*
Pulmonary hypertension (%)	12.8	13.9	0.9	0.108
Urgency (%)				0.001*
Elective	49.8	53.8	39.9	
Urgent	48.8	45.3	57.5	
Emergency	1.4	0.9	2.6	
Weight of intervention (%)				0.091
Isolated CABG	68.9	67.1	73.4	
Single non CABG	13.1	13.9	11.2	
2 procedures	15.9	16.9	13.3	
3 procedures	2.1	2.1	2.1	
Surgery on thoracic aorta (%)	2.7	3.1	1.7	0.262
**Intraoperative characteristics**				
Aortic cross-clamp (min)	60 [42-83]	60 [41-84.25]	61 [44-78]	0.879
ECC (min)	91 [63-118]	90 [62.75-119]	91 [65.5-116]	0.908
**Postoperative characteristics**				
Infection (%)	5.2	4.5	6.9	0.176
First detubation (hour)	3.2 [2.5-4.7]	3.2 [2.5-4.6]	3.2 [2.6-4.9]	0.485
Re-sternotomy <30d (%)	4.2	3.8	5.2	0.403
ICU days (n)	1.0 [1.0-1.0]	1.0 [1.0-1.0]	1.0 [1.0-1.0]	0.094
ICU extended (%)	5.8	5.4	6.9	0.420
CVA (%)	0.4	0.0	1.3	0.006*
Readmission ICU (%)	0.9	0.7	1.3	0.412
Readmission <30 days after discharge (%)	0.9	0.9	0.9	0.962
MI (excl. perioperative MI) (%)	0.7	0.5	1.3	0.938
MI (incl. Perioperative MI) (%)	3.0	2.4	4.3	0.215
PCI <1yr (%)	2.2	1.7	3.4	0.140

Abbreviations: R, recovery group; NR, non-recovery group; CVA, cerebral vascular accident; AP, angina pectoris; EuroSCORE, European System for Cardiac Operative Risk Evaluation; NYHA, New York Heart Association; CCS Class 4 Angina, Inability to perform any activity without angina or angina at rest; LVEF, left ventricular ejection fraction; MI, Myocardial infarction; CABG, coronary artery bypass grafting; ECC, extracorporeal circulation; ICU, intensive care unit; PCI, percutaneous coronary intervention; *Indicates a significant difference across groups. Data are presented as median [interquartile range] unless stated otherwise.

ESM Table 2 . Multivariate analysis of all cardiac surgery patients. Dependent variable: mental recovery.

	p-value	OR	95% CI
Baseline SF36 mental domain score	<0.001	0.953	0.943-0.963
Recent MI	0.006	0.587	0.402-0.856
Infection	0.027	0.452	0.224-0.914
PCI < 1 year	0.017	0.270	0.092-0.793

Hosmer and Lemeshow X^2=13.889, p=0.085; Nagelkerke R^2=0.192

ESM Table 3. Characteristics of isolated CABG patients in the mental recovery group and non-recovery group

	All (n=553)	R (n=381)	NR (n=172)	p-value
**Demographics**				
Baseline SF 36 mental domain score	70 [55-82]	66 [52-77]	79 [65-90]	<0.001*
Body Mass Index (kg/m²)	27.1 [24.8-29.6]	26.9 [24.8-29.4]	27.4 [24.8-29.7]	0.512
**Comorbidities**				
CVA (%)	0.7	0.8	0.6	0.791
Neurologic dysfunction (%)	1.1	1.0	1.2	0.906
**Cardiac status**				
Unstable AP (%)	4.9	4.7	5.2	0.400
**EuroSCORE II**	1.37 [0.98-2.11]	1.3 [0.9-2.0]	1.5 [1.1-2.5]	0.537
Age (years)	68.4 [61.8-74.1]	68.6 [61.9-74.3]	68.3 [61.8-73.8]	0.574
Gender male (%)	83.2	85.8	77.3	0.013*
Serum Creatinine (umol/l)	84.0 [74.5-95.0]	85.0 [76.0-94.5]	82.0 [72.0-96.0]	0.150
Extracardiac arteriopathy (%)	7.8	7.1	9.3	0.368
Poor Mobility (%)	1.1	1.0	1.2	0.906
Previous cardiac surgery (%)	1.4	1.0	2.3	0.245
Chronic lung Disease (%)	10.1	10.8	8.7	0.462
Active endocarditis (%)	0.0	0.0	0.0	1.000
Critical preoperative state (%)	0.5	0.5	0.6	0.933
Diabetes (%)	19.7	18.9	21.5	0.475
NYHA Class III or IV (%)	57.1	54.1	64.0	0.030
Angina CCS Class IV (%)	2.4	2.4	2.3	0.979
LVEF (%)				0.448
Good	79.7	80.1	76.2	
Moderate	17.6	16.3	19.8	
Poor	2.7	2.4	3.5	
missing		1.3	0.6	
Recent MI (%)	29.5	24.4	40.7	<0.001*
Pulmonary hypertension (%)	1.8	2.1	1.2	0.444
Urgency (%)				0.007*
Elective	38.5	42.5	29.7	
Urgent	59.9	56.4	67.4	
Emergency	1.6	1.0	2.9	
Weight of intervention (%)				0.795
Isolated CABG	99.3	99.2	99.4	
Single non CABG	0.0	0.0	0.0	
2 procedures	0.5	0.5	0.6	
3 procedures	0.2	0.3	0.0	
Surgery thoracic. Aorta (%)	0.2	0.0	0.6	0.137
**Intraoperative characteristics**				
Aortic cross-clamp (min)	51.0 [37.0-70.0]	50.0 [36.0-69.5]	52.0 [39.0-71.0]	0.252
ECC (min)	80.0 [55.0-103.0]	78.0 [53.0-103.0]	84.0 [57. -105.0]	0.154
**Postoperative characteristics**				
Infection (%)	5.1	4.5	6.4	0.337
First detubation (hour)	3.0 [2.5-4.4]	3.08 [2.5-4.3]	3.0 [2.6-4.5]	0.634
Re-sternotomy<30d (%)	3.8	4.2	2.9	0.746
ICU days (n)	1.0 [1.0-1.0]	1.0 [1.0-1.0]	1.0 [1.0-1.0]	0.850
ICU stay extended (%)	1.5	2.6	2.9	0.850
CVA (%)	0.2	0.0	0.6	0.136
Readmission ICU (%)	0.5	0.5	0.6	0.933
Readmission <30 days after discharge (%)	1.1	1.1	1.2	0.937
MI (excl. perioperative) (%)	1.1	0.6	2.0	0.376
MI (incl. perioperative (%)		2.6	5.8	0.251
PCI <1yr (%)	2.9	2.1	4.7	0.098

Abbreviations: R, recovery group; NR, non-recovery group; CVA, cerebral vascular accident; AP, angina pectoris; EuroSCORE, European System for Cardiac Operative Risk Evaluation; NYHA, New York Heart Association; CCS Class 4 Angina, Inability to perform any activity without angina or angina at rest; LVEF, left ventricular ejection fraction; MI, Myocardial infarction; CABG, coronary artery bypass grafting; ECC, extracorporeal circulation; ICU, intensive care unit; PCI, percutaneous coronary intervention; *Indicates a significant difference across groups. Data are presented as median [interquartile range] unless stated otherwise.

ESM Table 4. Multivariate analysis of isolated CABG patients. Dependent variable: mental recovery.

	p-value	OR	95% CI
Baseline SF 36 domain score mental	<0.001	0.953	0.914-0.966
Female sex	<0.001	0.383	0.228-0.644
Previous cardiac surgery	0.028	0.185	0.041-0.838
Recent MI	0.004	0.537	0.352-0.815
PCI < 1 year	0.016	0.245	0.078-0.770

Hosmer and Lemeshow X^2=7.939, p=0.439; Nagelkerke R^2=0.212

EMS Table 5. Characteristics of all cardiac surgery patients in the Responders and non-Responders group.

	Responders (n=803)	Non-Responders (n=970)	p-value
**Baseline characteristics**			
Baseline SF 36 physical domain score	59 [45-73]		
Baseline SF 36 mental domain score	68 [54-81]		
Body Mass Index (kg/m^2)	27 [25-30]	27 [24-30]	0.685
CVA (%)	1.5	2.1	0.372
Neurologic dysfunction (%)	2.0	4.0	0.014*
Unstable AP (%)	3.5	5.3	0.072
**EuroSCORE II**	1.6 [1.1-2.7]	1.9 [1.2-3.5]	<0.001*
Age (years)	69 [62-75]	69 [62-76]	0.773
Gender Male (%)	76.8	70.5	0.003*
Serum Creatinine (umol/l)	84 [73-96]	82 [71-97]	0.145
Extracardiac arteriopathy (%)	7.8	12.0	0.004*
Poor mobility (%)	2.0	4.0	0.014*
Previous cardiac surgery (%)	3.1	3.9	0.363
Chronic lung disease (%)	11.5	12.4	0.555
Active endocarditis (%)	0.7	2.0	0.031*
Critical preoperative state (%)	0.5	2.5	0.001*
Diabetes (%)	18.9	21.8	0.143
NYHA Class III or IV (%)	53.8	62.8	<0.001*
Angina CCS Class IV (%)	1.9	3.2	0.080
LVEF (%)			0.016*
Good	77.1	72.5	
Moderate	18.6	20.2	
Poor	4.4	7..3	
Recent MI (%)	21.4	24.4	0.134
Pulmonary hypertension (%)	12.8	17.5	0.006*
Urgency (%)			<0.004*
Elective	50.1	43.8	
Urgent	48.6	55.4	
Emergency	1.4	0.8	
Weight of intervention (%)			0.092
Isolated CABG	69.4	66.9	
Single Non CABG	13.1	12.1	
2 Procedures	15.4	17.0	
3 Procedures	2.1	4.0	
Surgery on thoracic aorta (%)	2.7	3.8	0.209
**Intraoperative characteristics**			
Aortic cross-clamp (min)	60 [42-83]	60 [41-84]	0.641
ECC (min)	91 [64-118]	90 [64-120]	0.847
**Postoperative characteristics**			
Infection (%)	5.6	7.1	0.197
First detubation (hour)	3.2 [2.5-4.7]	3.1 [2.5-4.6]	0.333
Re-sternotomy <30 d (%)	4.1	7.3	0.003*
ICU days (n)	1.0 [1.0-1.0]	1.0 [1.0-1.0]	0.037*
ICU stay extended (%)	6.0	10.6	<0.001*
CVA (%)	0.2	1.0	0.046*
Readmission ICU (%)	1.0	2.1	0.073
Readmission <30days after discharge (%)	1.1	2.0	0.153
MI (incl. perioperative MI) (valid%)	3.4	4.5	0.111
MI (excl. perioperative MI) (valid%)	0.9	1.2	0.361
PCI <1year (%)	2.2	3.0	0.329

Abbreviations: CVA, cerebral vascular accident; AP, angina pectoris; EuroSCORE, European System for Cardiac Operative Risk Evaluation; NYHA, New York Heart Association; CCS Class 4 Angina, Inability to perform any activity without angina or angina at rest; LVEF, left ventricular ejection fraction; MI, Myocardial infarction; CABG, coronary artery bypass grafting; ECC, extracorporeal circulation; ICU, intensive care unit; PCI, percutaneous coronary intervention; *Indicates a significant difference across groups. Data are presented as median [interquartile range] unless stated otherwise.

ESM Figure 1. Flow chart of CABG patients.

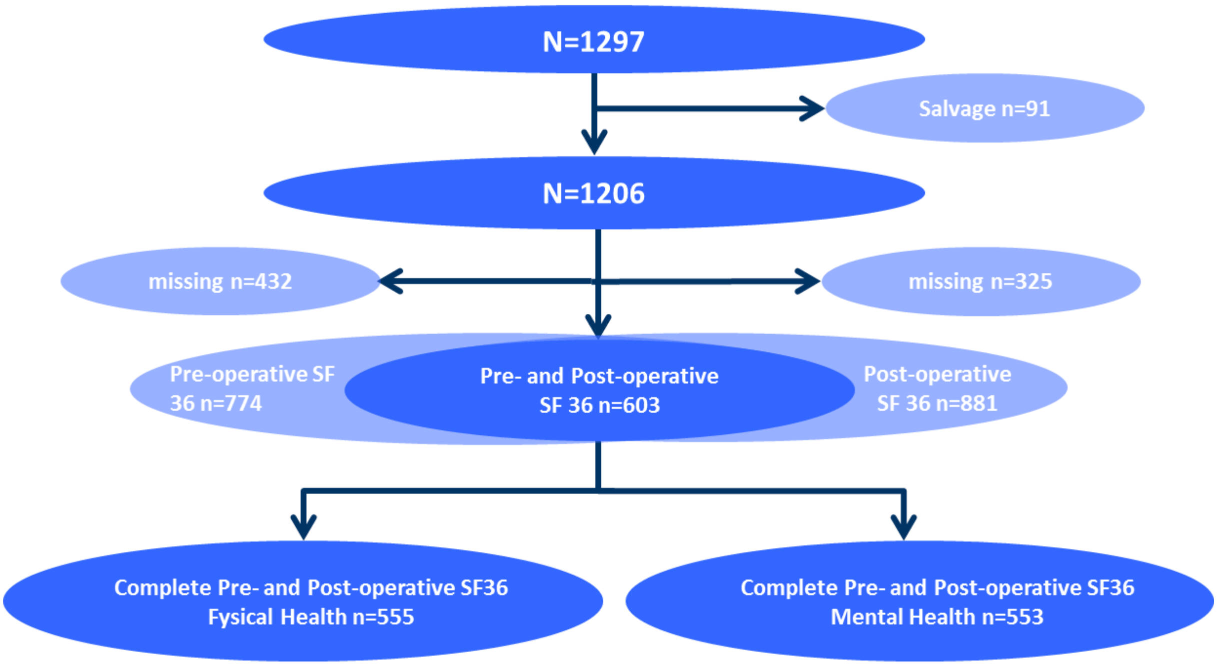

ESM Figure 2. Venn diagram of all patients. Interplay between changes in SF-36 physical and mental domain scores prior to and 1 year after surgery (%).

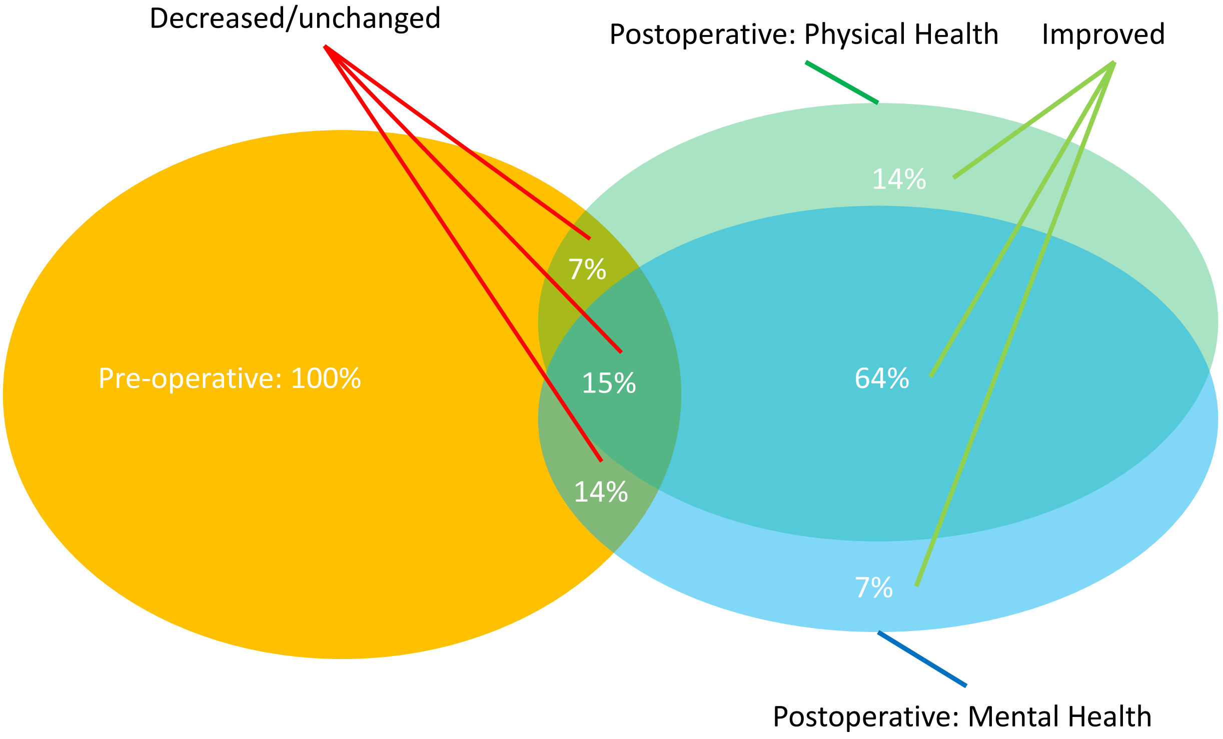

ESM Figure 3. Venn diagram of isolated CABG patients. Interplay between changes in SF-36 physical and mental domain scores prior to and 1 year after surgery.

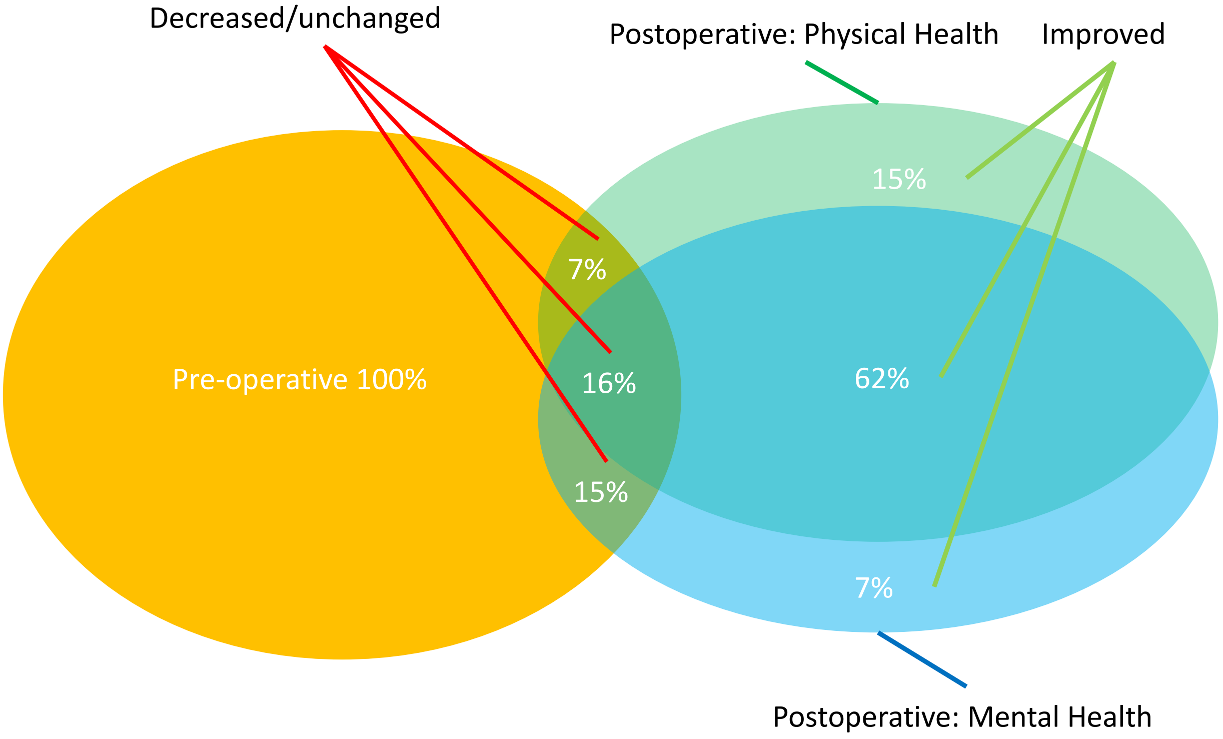

Supplement: Supplementary file 1 — Additional file 1 Table 1. Characteristics of all cardiac surgery patients in the mental recovery group and non-recovery group. Table 2. Multivariate analysis of all cardiac surgery patients. Dependent variable: mental recovery. Table 3. Characteristics of isolated CABG patients in the mental recovery group and non-recovery group. Table 4. Multivariate analysis of isolated CABG patients. Dependent variable: mental recovery. Table 5. Characteristics of all cardiac surgery patients in the Responders and non-Responders group. Figure 1. Flow chart of CABG patients. Figure 2. Venn diagram of all patients. Interplay between changes in SF-36 physical and mental domain scores prior to and 1 year after surgery (%). Figure 3. Venn diagram of isolated CABG patients. Interplay between changes in SF-36 physical and mental domain scores prior to and 1 year after surgery. [file 13019_2020_1273_MOESM1_ESM.pdf]
